# Reduction of membrane fouling by innovative method (injection of air jet)

**DOI:** 10.1186/s40201-014-0128-0

**Published:** 2014-11-14

**Authors:** Maryam-Sadat Amiraftabi, Navid Mostoufi, Mostafa Hosseinzadeh, Mohammad-Reza Mehrnia

**Affiliations:** School of Chemical Engineering, College of Engineering, University of Tehran, Tehran, Iran

**Keywords:** Membrane bioreactor, Fouling control, Shear stress, CFD, Jet injection

## Abstract

**Background:**

One of the most important challenges about the Membrane Bio Reactors is membrane fouling. Fouling has been at the centre of a globe debate for more recent years. It leads to high operational and maintenance costs such as membrane damage and replacement of membrane. Membrane fouling is attributed to the physicochemical interactions between the bio fluid and membrane. In order to decrease the fouling in bioreactors there are common anti fouling strategies such as operation at low flux, Optimization of aeration flow-rate and Physical and chemical cleanings. However, often they are not effective.

**Methodology:**

This work deal with fouling crisis by a new and innovative method in order to reduce of fouling on membrane surface by injection of parallel air jet on membrane bio reactor. This is a new idea and fundamental study about the influence of wall jet on fouling of membrane surface. This study is included both experimental and numerical investigations. In order to polarize the stream path on the surface of the membrane, four symmetric nozzles were implemented at the bottom of the membrane surface upon the sparger. The changes in the fouling resistance were experimentally measured at five various jet velocities and all of them recorded by a computer system. In addition the effect of air jet velocity and shear stress on fouling resistances was also investigated by computational fluid dynamics at the similar conditions.

**Results:**

It was revealed that the permeate flux and resistance of fouling can be related to shear stress of air flow at the membrane surface. When the velocity of air jets increase, the permeate flux increase too. Also, results illustrate that jet injection can partially remove the cake which was formed on the surface of the membrane.

**Conclusions:**

Correlations were developed for estimating each resistance of the membrane surface via the shear stress. The resistances of the cake are removed by the jet velocity changes, from 20% in lower jet velocity up to 40% in higher jet velocity.

## Background

Submerged membrane bioreactors (SMBRs) are widely used in wastewater treatment. One of the SMBR types is airlift membrane bioreactor (AMBRs) which contains two baffles that separate the bioreactor into a riser and two downcomers. Although the downcomer is filled mainly from the liquid, but the riser is gassed. As a result of the density difference between the bubbly mixture in the riser and the liquid in the downcomer, the flow circulates between these two sections [1].

The hydrodynamic properties and mixing pattern in MBRs depend on many factors, such as gas properties, liquid properties, gas entrance conditions and distributor geometry. Complexity of the hydrodynamics and development of efficient numerical methods have led researchers to employ computational fluid dynamics (CFD) to study the hydrodynamics of two-phase flow in MBRs [2–7].

The MBR filtration performance inevitably decreases with filtration time. This is due to the deposition of soluble and particulate materials onto and into the membrane, attributed to the interactions between activated sludge components and the membrane. This major drawback and process limitation has been under investigation since the early MBRs, and remains one of the most challenging issues facing further MBR development [8].

In recent reviews covering membrane applications to bioreactors, it has been shown that, as with other membrane separation processes, membrane fouling is the most serious problem affecting system performance. Fouling leads to a significant increase in hydraulic resistance, manifested as permeate flux decline or transmembrane pressure (TMP) increase when the process is operated under constant-TMP or constant-flux conditions respectively. In systems where flux is maintained by increasing TMP, the energy required to achieve filtration increases. Alternatively frequent membrane cleaning is therefore required, increasing significantly the operating costs as a result of cleaning agents and production downtime. More frequent membrane replacement is also expected [8].

A usual method to reduce the fouling on the membrane is to put a sparger below the membrane surface to inject air near the surface of the membrane and removing the fouling through the shear stress exerted on the surface. Some researchers have studied the effect of aeration on the membrane fouling and filtration of waste water [9–14]. According to these researches, the shear stress, generated by the aeration, has a large effect on reduction of filtration resistance in the SMBR. Increasing the permeate flux in a two-phases MBR is due to enhancement of the shear stress on the membrane surface [15]. Injection of gas increases the turbulence, which reinforces the shear stress [10,16]. This strategy was shown to be very effective for flux enhancement in different membrane processes, in particular microfiltration [17,18], ultrafiltration [10,19] and nanofiltration [18] as well as for different membrane geometries such as tubular [10,16,17,20], hollow fibre [17,19,20] and flat-sheet modules [17,18].

In order to illustrate the results of workout wall jets, the effect of shear stress which is produced by flow of gas on the membrane surface at various conditions should be known accurately. Many researchers have measured the shear stress at the surface of the membrane in the liquid phase by using electrochemical method [9,21]. Computational fluid dynamics (CFD) is a technique which solves the equations of motion and overcomes some disadvantages of other methods. By the CFD method the opportunity for analyzing the effect of geometry/configuration of bioreactors and hydrodynamics of the flows can also be provided. The objective of this work is to understand the effect of wall shear stress on the cake filtration resistance and finding the better way to decrease of fouling on membrane surface. To reach this goal, filtration experiments and CFD numerical simulation were performed on the same conditions of the MBR.

## Methods

### Experiments

#### Experimental set-up

**Figure 1:**
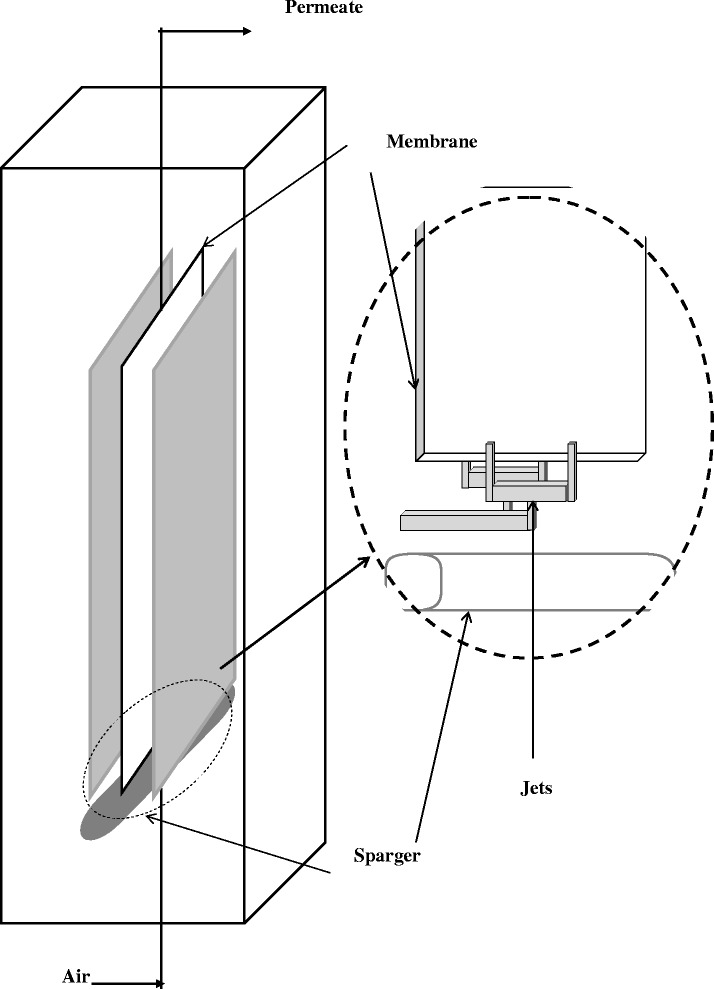
**Scheme of set up.**

The experiments were carried out in an airlift SMBR (H × W × D = 70 cm × 24 cm × 18 cm) that is shown in Figure 1, here D is the Depth of reactor, W is the width of reactor and H is the Height of reactor.

The reactor had 22 litre capacities; it contained two baffles (31 cm high and 24 cm width) which divided the bioreactor. It involved a riser and two downcomers. In the middle of the MBR, a flat sheet membrane module, made by KUBOTA Co. (Japan) with a mean pore size of 0.45 μm, was installed vertically which is located between the two baffles. Effective filtration area was 0.116 m^2^. A gas sparger was placed under the membrane for aeration. The gas sparger was a flexible porous rubber (3 cm × 21 cm) with 25 holes/cm^2^. In addition four nozzles were fixed at each side of the membrane which nozzle slots were 5.82 mm × 0.72 mm. They used to evoke the air jet for removing the fouling cack which was formed on membrane surface.

#### Materials

The activated sludge which was used in experiments was taken from the wastewater treatment plant of Tehran Oil Refining Co. (Iran). The trans-membrane pressure (TMP) was monitored by a pressure gauge and was kept constant at 0.4 bars during the experiments. The concentration of mixed liquor suspended solid (MLSS) of 10 ± 0.1 grL^−1^ was used in the experiments. Sludge retention time was about 20 days and the organic loading rate was 0.18–0.19 gCOD/gMLSS/day. Composition of the wastewater is presented in Table 1 and the main operating parameters are presented in Table 2. Each experiment was repeated twice to ensure the repeatability of the experimental results.

**Table 1:** **The composition of standard wastewater**

**Components**	**Concentration (mg/L)**
Glucose	1350
(NH_4_)_2_SO_4_	215
(NH_4_)H_2_PO_4_	38
MgSO_4_ · 7H_2_O	27.5
KCl	20
FeSO_4_ · 7H_2_O	2.5
NaHCO_3_	557.7

**Table 2:** **The operational parameters of the bench-scale airlift membrane bioreactor**

**Parameters**	**Unit**	**Average**
Flux	Lm^2^ h^−1^	16.4
TMP	bar	0.4
DO	mgO_2_ L^−1^	2.4
Temperature	°C	19.9
pH		6.9

#### Experiment procedure

The membrane flux was calculated by measuring the total weight of permeate which was leaving the membrane module. Permeate flux (*J*) is calculated by following equation:

1 $$ J=\frac{m\left|{}_{t+\Delta t}-m\right|{}_t}{A\Delta t} $$

Where m|t is the weight of total permeate flow at time t and A is membrane surface. Here ∆t is time increment (sec), J is value of membrane flux (Lm^−2^hr^−1^). This value was measured each 15 second by flux of passing purified water. When the permeate flux became constant, it was used for calculation of membrane resistance (Rm):

2 $$ {R}_m=\raisebox{1ex}{$\Delta P$}\!\left/ \!\raisebox{-1ex}{$\mu J$}\right. $$

P is pressure (Pa)

μ_J_ is permeate viscosity (mPas^−1^)

After membrane fouling flux approximately becomes constant, the resistance of fouling in this situation (*R*_*t*_), can be evaluated by the series model [22]:

3 $$ Rt=\frac{\Delta P}{\mu {J}_t}={R}_m+{R}_{pb}+{R}_c+{R}_j $$

*R*_*m*_ is resistance of clean membrane (m^−1^)

*R*_*j*_ is jet resistance (m^−1^)

R_t_ is total resistance (m^−1^)

*R*_*pb*_ is pore blockage resistance (m^−1^)

R_c_ is resistance of cake that couldn’t be removed by aeration and need to special physical washing (m^−1^)

*J*_*t*_ is the flux of water before cleaning (Lm^−2^hr^−1^*)*

In this equation, Rj is the resistance of membrane fouling that is reduced by jet injection. *J*_j_ is the flux of water after surface cleaning with jets (Lm^−2^hr^−1^*).*), *J*_m_ is value of flux of clean membrane (Lm^−2^hr^−1^)

4 $$ {R}_j={R}_t-\frac{\Delta P}{\mu {J}_j} $$

and *R*_c_ is the remaining resistance of fouling that can be cleaned by physical washing such as relaxation, backwashing or cleaning by sponge balls:

5 $$ {R}_c={R}_t-{R}_j-\frac{\Delta P}{\mu {J}_j} $$

The remaining resistance of fouling after physical washing is not equal to the resistance of the clean membrane (*R*_m_). This difference is called *R*_pb_

6 $$ {R}_{pb}=\frac{\Delta P}{\mu J}-{R}_m $$

This resistance occurs inside the membrane structure due to pore blockage and only chemical washing can affect it.

### Numerical simulations

The SMBR was simulated using a 3D, two-phase model by an Eulerian-Eulerian approach [22]. For modeling the turbulence, the standard *k*-ε model was used. The geometry of the SMBR was considered as the same as that used in the experiments. Only one quarter of the reactor was simulated. The best mesh was chosen by Richardson validation [23]. Mass and momentum balance equations solved for both phases are listed in Table 3.

**Table 3:** **Mass and momentum balance equations considered for the CFD modeling**

**Equation type**	**Equation**
**Continuity**	$\frac{\partial }{\partial t}{\propto}_L{\rho}_L+\nabla .\left({\propto}_L{\rho}_L{\overline{u}}_L\right)=0$
	$$ \frac{\partial }{\partial t}{\propto}_G{\rho}_G+\nabla .\left({\propto}_G{\rho}_G{\overline{u}}_G\right)\kern0.5em =0 $$
***Momentum balance***	$\frac{\partial }{\partial t}\left({\propto}_L{\rho}_L{\overline{u}}_L\right)+\nabla .\left({\propto}_L{\rho}_L{\overline{u}}_L{\overline{u}}_L\right)=-{\propto}_L\nabla P+\nabla .\;{\overline{\overline{\tau}}}_L+{K}_{GL}\left({\overline{u}}_G-{\overline{u}}_L\right)+{\propto}_L{\rho}_L\overline{g}$
	$$ \frac{\partial }{\partial t}\left({\propto}_G{\rho}_G{\overline{u}}_G\right)+\nabla .\left({\propto}_G{\rho}_G{\overline{u}}_G{\overline{u}}_G\right)=-{\propto}_G\nabla P+\nabla .{\overline{\overline{\tau}}}_G+{K}_{GL}\left({\overline{u}}_L-{\overline{u}}_G\right)+{\propto}_G{\rho}_G\overline{g} $$
**Standard** ***k*** **–** ***ε*** **model**	$\frac{\partial }{\partial t}{\rho}_mk+\nabla .{\rho}_m{\overline{u}}_mk=\nabla .\left(\frac{{\mu^t}_m}{\sigma k}\nabla k\right)+{G}_{k,m}-{\rho}_m\varepsilon$
	$$ \frac{\partial }{\partial t}{\rho}_m\varepsilon +\nabla .{\rho}_m{\overline{u}}_m\varepsilon =\nabla .\left(\frac{{\mu^t}_m}{\sigma_{\varepsilon }}\nabla \varepsilon \right)+\frac{\varepsilon }{k}\left({C}_{1\varepsilon }{G}_{k,m}-{C}_{2\varepsilon }{\rho}_m\varepsilon \right) $$
**Mixture properties**	*ρ*_*m*_ = ∝ _*G*_ *ρ*_*G*_ + ∝ _*L*_ *ρ*_*L*_
	$$ {\overline{u}}_m=\frac{\propto_G{\rho}_G{\overline{u}}_G+{\propto}_L{\rho}_L{\overline{u}}_L}{\rho_m} $$
	$$ {\mu^t}_m={\rho}_m{C}_{\mu}\frac{k^2}{\varepsilon } $$

In which C_1ε_ =1.44 and C_2ε_ =1.92 and C_μ_ =0.09 [22].

*α is* void fraction

*α*_*i*_*is* volume fraction of phase i

*ε is* turbulent dissipation rate (m^2^s^−3^)

*ρ*_*i*_*is* density of phase i (kgm^−3^)

*v* is jet velocity (ms^−1^)

*u*_*i*_ is velocity of phase i (ms^−1^)

*g* is gas phase

*l* is liquid phase

*C*_*1ε*_ is constant

*C*_*2ε*_ is constant

*C*_*μ*_ is constant

g_c_ is gravitation acceleration, 9.81 ms^−2^

*G* is velocity gradient, s^−1^

*k* is turbulent kinetic energy, m^2^s^−2^

### Turbulent jet

In the present work, it was considered that the jet comes out of a slit as a turbulent jet. The geometry and flow of the boundary layers in the simulation were considered to be as the same as in the experiments. The membrane was modeled as a wall since permeate flux from the membrane is negligible compared to the jet flow. Time averaged Navier–Stokes equation for the turbulent boundary layer is:

7 $$ \overline{u}\kern0.5em \frac{\partial \overline{u}}{\partial x}+\overline{v}\frac{\partial \overline{u}}{\partial y}=-\frac{1}{\rho}\frac{\partial \overline{p}}{\partial x}+v\frac{\partial^2\overline{u}}{\partial {y}^2}-\frac{\partial }{\partial y}\left(\overline{u^{\prime }v^{\prime }}\right) $$

8 $$ \overline{u}\kern0.5em \frac{\partial \overline{v}}{\partial x}+\overline{v}\frac{\partial \overline{v}}{\partial y}=-\frac{1}{\rho}\frac{\partial \overline{p}}{\partial x}+v\frac{\partial^2\overline{v}}{\partial {y}^2}-\frac{\partial }{\partial y}\left(\overline{v{\prime}^2}\right) $$

According to the above Reynolds-averaged momentum equations, the diffusion terms and derivative of Reynolds stresses in the axial direction were neglected [24]. Also, mean velocity field satisfies the following continuity equation:

9 $$ \frac{\partial \overline{u}}{\partial x}+\frac{\partial \overline{v}}{\partial y}=0 $$

Here *u*′ is fluctuation of turbulent shear stresses and *v*′ is fluctuation of turbulent shear stresses also $\left(\overline{u^{\prime }v^{\prime }}\right)$ is flow variables or derivatives.

For finding the best solution of the turbulent boundary layer equations, the use of a turbulence model is necessary. For this purpose, the shear stresses or turbulent stresses were modelled by the *k*-ε model. It is worth mentioning that other turbulence models were also tried and it was found that *k*-ε is the best for simulation of the jet.

### *k*-ε model

The most appropriate turbulence models are two-equation models in which solution of two separate transport equations allows the turbulent velocity and length scales to be independently determined. The standard *k*-ε model is a semi-empirical model based on transport equation model for the turbulence kinetic energy (*k*) and its dissipation rate (ε).

10 $$ \overline{ui}\kern0.5em \frac{\partial k}{\partial {x}_i}=\frac{\partial }{\partial {x}_i}\left(\frac{v_t\kern0.5em }{\delta k}\frac{\delta k}{\delta {x}_i}\right)+p+{g}_c-\varepsilon $$

11 $$ \overline{u_i}\frac{\partial \varepsilon }{\partial {x}_i}=\frac{\partial }{\partial {x}_i}\left(\frac{v_t}{\delta \varepsilon}\frac{\delta \varepsilon }{\delta {x}_i}\right)+{c}_{\varepsilon_1}\frac{\varepsilon }{k}p-{c}_{\varepsilon_2}\frac{\varepsilon^2}{k}+{g}_c\frac{\varepsilon }{k} $$

The transport equation model for *k* is derived from exact equation, while the transport equation model for ε was obtained using physical reasons [25].

## Results and discussions

**Figure 2:**
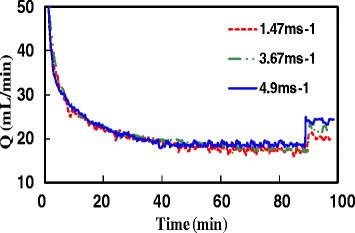
**Permeate flux increase after 10 second jet injection at (25, 5 and 15 mLmin** ^**−1**^ **in t = 90 min), the injected jet velocities are (1.47, 3.67, and 4.9 ms** ^**−1**^ **).**

Various permeate flows vs. time are shown in Figure 2. This figure demonstrates that approximately 45 minutes after start of the operation; permeate flow leveled off as little as 20mLmin^−1^ because of the creation of fouling on the membrane. At time *t* =90 min., air jet was injected with various velocities of 1.47, 3.67 and 4.9 m/s. The jet was injected when the bioreactor was in steady state situation and the permeate flux is in its minimum. Figure 2 shows that when the jet is injected with 1.47 m/s, the permeate flow is improved up to 20%. In fact, the injection of jet generates shear stress on the membrane surface. Therefore it leads to remove the cake from the membrane surface and decrease the cake resistance. In the other words, the jet has a positive effect on removing the membrane fouling. Figure 2 also illustrates that the higher velocity of jet, the higher permeate flow (corresponding to higher removal of the cake).

**Figure 3:**
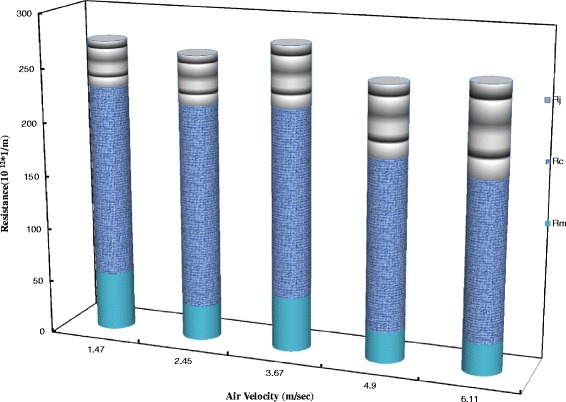
**The various resistances vs. jet Velocities by experimental data.**

In each experiment, there is a total resistance which related to the fouling resistance before jets injection. Figure 3 shows various resistances vs. jet velocity. This figure reveals that resistance of the clean membrane is almost equal in all cases, it conforms that all these tests have been started at the same conditions. Nevertheless, this resistance is only about 5% of the total resistance. Figure 3 also demonstrates that when the jet velocity is increased, the resistance removed by the jet (hashed zone, *R*_j_) increased and the cake resistance decreased. In other words, the resistance of the remaining cake on the membrane surface (*R*_c_) is decreased. Also, the amount of removed cake is increased by raising the jet velocity.

**Figure 4:**
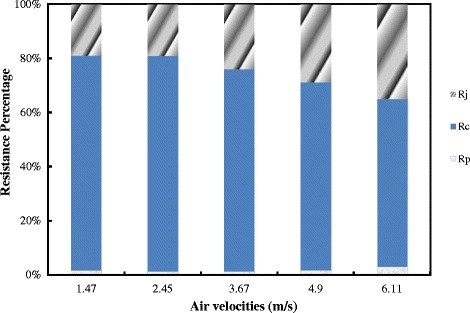
**The percentage of different resistance vs. air fluxes of jets by experimental.**

The percentage of reduction in the cake resistance due to jet injection is shown in Figure 4. This figure illustrates that jet injection can partially remove the cake which formed on the surface of the membrane. It means, it can be used as a strategy to improve filtration performance in the MBR. However, by increasing the air velocity, the percentage of the removed cake is increase. In fact, when the jet is injected, the shear stress increases on the surface of the membrane which can remove the fouling. The resistances of the cake are removed by the jet changes from 20% in lower jet velocity up to 40% in higher jet velocity. According to Figure 4, when the jet velocity is increased, the permeate flux rose significantly up to 40%. By increasing the jet velocity, the shear stress on the membrane grew which lead to fall in the cake resistance. It is so important to notice that, the shear stress causes separate of colloids and removed absorbed particles from the membrane surface.

**Figure 5:**
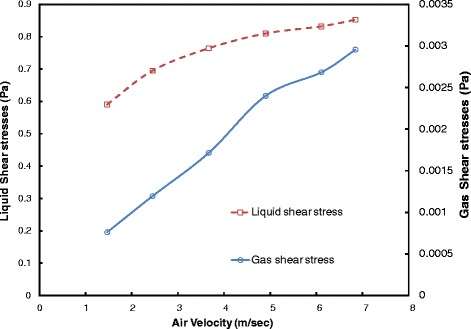
**The total shear stresses vs. air velocity of jets by numerical simulation.**

Results of the shear stress in CFD simulation are as the same as the experiments which are shown in Figure 5. As can be seen in this figure, the average shear intensity on the surface of the membrane has been increased by increasing the air flux*.* Figure 5 illustrates that the shear stress on the surface of membrane which is produced by flow of air, is two orders of magnitude smaller than that was in the liquid. However, changing in the shear stress of air flow is sharper than the liquid. The mainly part of removing the cake from the membrane surface, is for liquid shear stress. Although this huge amount of shear stresses on liquid is because of injection of air jet in liquid. In fact, large sludge particles and accumulated on the membrane surface, can be removed by the shear stress which were generated by air flow.

Figure 5 also demonstrates that mean shear stresses of both liquid and gas have linear function of the jet entrance velocity. The following correlations can be proposed for evaluating these shear stresses based on the jet velocity:

12 $$ {\tau}_g=0.4135{V}_j+0.1983 $$

13 $$ {\tau}_L=0.0452{V}_j+0.5658 $$

τ is shear stress (Nm^−2^)

*τ*_*i*_ is stress tensor of phase i(Nm^−2^)

The cake resistance can be a multiple linear function consistent of shear stresses of both gas and liquid phases. In the experimental work, resistance fall by increasing shear stresses, thus, it can be assumed that the total resistance is a linear function of shear stresses of both gas and liquid. In order to find a proper equation, the following general multi-linear equation was considered:

14 $$ {R}_t=A+B{\tau}_g+C{\tau}_L+\mathrm{D}{\tau_{\mathrm{g}}}^2+E{\tau_L}^2+F{\tau}_L{\tau}_g $$

The coefficients of this multiple linear equation calculated according to the experimental data by using the multiple linear regressions and the least square technique [26]. The equation was then simplified using the method of analysis of variances (ANOVA) by eliminating the insignificant terms. The final result is:

15 $$ {R}_t=-1033.31+59991.46{\tau}_g+3765.43{\tau}_L-3037.76{\tau_L}^2 $$

**Figure 6:**
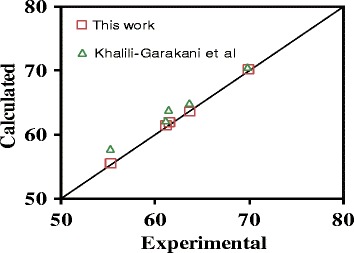
**Comparison of the cake resistances vs. air velocity of jets between experimental results and correlation.**

The correlation coefficient of this equation was calculated to be 0.999. Figure 6 demonstrates a comparison between the calculated and experimental resistances. The cake resistances calculated by the correlation of [22] are also shown in this figure. Figure 6 illustrates that there is a good agreement between the experimental data and cake resistances predicted by Eq. (8). Also, the correlation developed in this work is better than proposed by [22] since their work was carried out in an air lift reactor whose hydrodynamics is slightly different than that considered in this work.

## Conclusions

On the whole, membrane fouling is a controversial issue and it always leads lots of costs. Flux reduction occurs because of membrane fouling and formation of cake on the membrane surface. In this study, it is possible to remove part of fouling by using the air jets, which leads to the increasing in the permeate flux. By imposing proper shear stress on the surface of the membrane through jets of air, the cake can be removed and fouling reduced. In addition, the resistances were determined experimentally, also, the shear stresses on the membrane surface for air and sludge were evaluated by CFD simulation. It was shown that there is an acceptable correlation between the resistance and shear stress. It was shown that, higher velocity of air passing across the membrane causes more shear stress on the surface and incredibly leads to improvement of cleaning process.
